# A ^14^C-leucine absorption, distribution, metabolism and excretion (ADME) study in adult Sprague–Dawley rat reveals β-hydroxy-β-methylbutyrate as a metabolite

**DOI:** 10.1007/s00726-015-1920-6

**Published:** 2015-01-25

**Authors:** Anthony J. Lee, David W. A. Beno, Xiaolin Zhang, Robin Shapiro, Mark Mason, Tanita Mason-Bright, Bruce Surber, Neilé K. Edens

**Affiliations:** 1 Drug Metabolism and Pharmacokinetics, AbbVie Inc., 1 North Waukegan Road, North Chicago, IL 60064 USA; 2 Strategic Research, Abbott Nutrition Division of Abbott Laboratories, 3300 Stelzer Road, Columbus, OH 43219 USA; 3 Process Organic Chemistry, AbbVie Inc., 1 North Waukegan Road, North Chicago, IL 60064 USA

**Keywords:** ADME, Leucine, Metabolism, HMB, β-Hydroxy-β-methylbutyrate, Branched-chain amino acid

## Abstract

Leucine is an essential branched-chain amino acid that acts as a substrate for protein synthesis and as a signaling molecule. Leucine not incorporated into muscle protein is ultimately oxidized through intermediates such as β-hydroxy-β-methylbutyrate (HMB) which itself is reported to enhance muscle mass and function in rats and humans. HMB has been reported in the plasma following oral leucine administration in sheep and pigs but not in Sprague–Dawley rats, the standard preclinical model. Therefore, we conducted radiolabeled absorption, distribution, metabolism and excretion (ADME) studies in rats using a low (3 mg/kg) or high dose (1,000 mg/kg) of ^14^C-leucine. Blood, tissue, and urine samples were analyzed for ^14^C-leucine and its metabolites by HPLC–MS. Our results show for the first time that ^14^C-HMB appears in plasma and urine of rats following an oral dose of ^14^C-leucine. ^14^C-leucine appears in plasma as ^14^C-α-ketoisocaproic acid (KIC) with a slower time course than ^14^C-HMB, a putative product of KIC. Further, two novel metabolites of leucine were detected in urine, *N*-acetyl leucine and glycyl leucine. Mass balance studies demonstrate that excretory routes accounted for no more than 0.9 % of the radiolabel and approximately 61 % of the dose was recovered in the carcass. Approximately 65 % of the dose was recovered in total, suggesting that approximately one-third of the leucine dose is oxidized to CO_2_. In conclusion, this study demonstrates endogenous production of HMB from leucine in adult rats, a standard preclinical model used to guide design of clinical trials in nutrition.

## Introduction

**Fig. 1:**
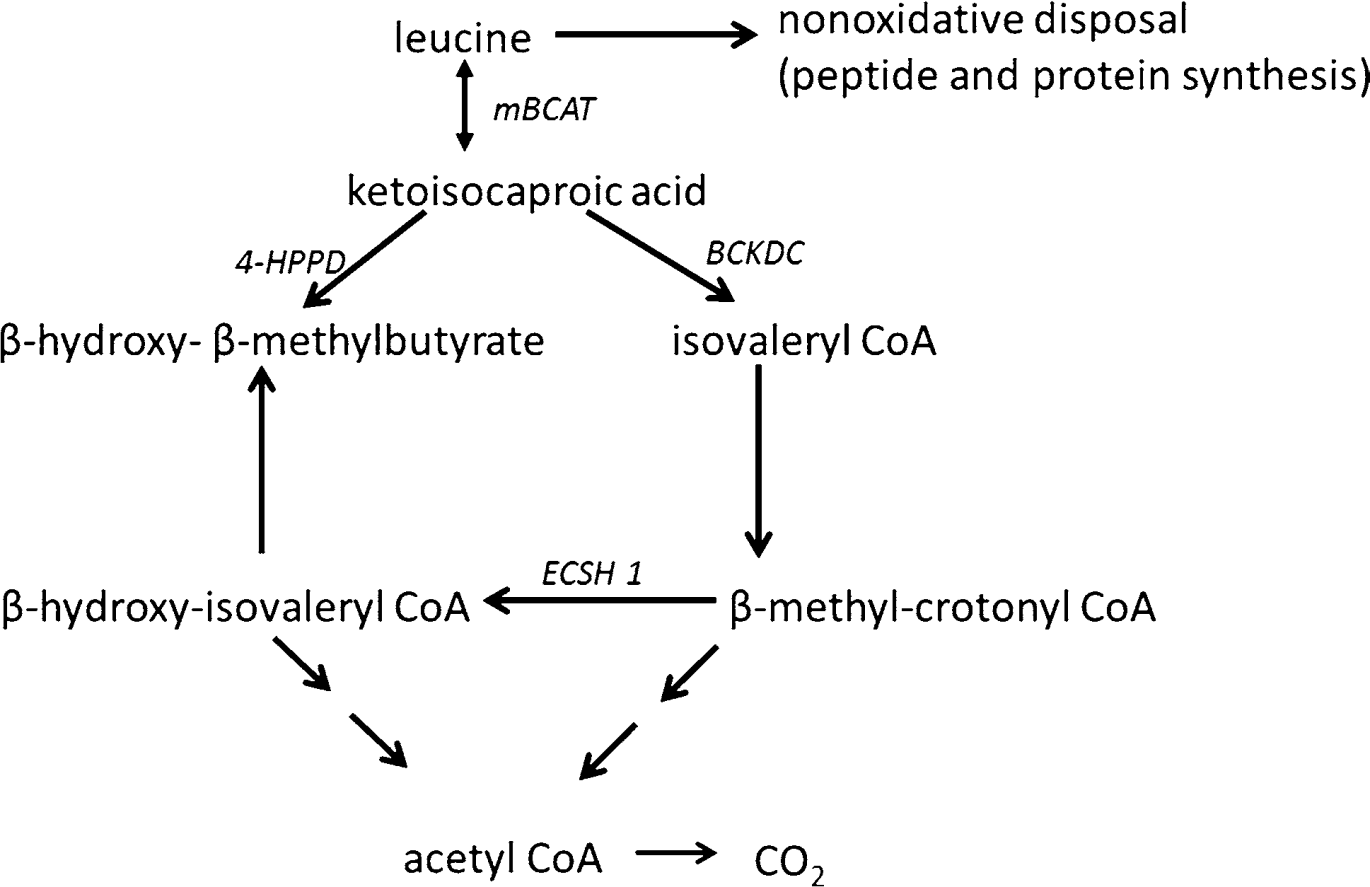
Metabolism of leucine in mammals. *mBCAT* mitochondrial branched-chain aminotransferase, *BCKDC* branched-chain ketoacid dehydrogenase complex, *4-HPPD* 4-hydroxy-phenylpyruvatedioxygenase, *ECSH1* short-chain enoyl-CoA hydratase. The first intermediate molecule yielded by leucine metabolism, α-ketoisocaproic acid (KIC), is formed when leucine is reversibly transaminated within the muscle by mitochondrial branched-chain amino transferase (mBCAT). Alternatively, a fraction of KIC is decarboxylated and reduced in liver to yield β-hydroxy-β-methylbutyrate (HMB), also known as β-hydroxyisovalerate (CAS 625-08-1). This reaction is catalyzed by ketoisocaproate dioxygenase (4-hydroxyphenylpyruvate dioxygenase; 4-HPPD; EC 1.13.11.27; CAS 9029-72-5) which has been detected in the livers of rat (Sabourin and Bieber 1982) and humans. Under some conditions, HMB can also be formed from leucine by the “ESCH 1 pathway”, which involves dehydrogenation of isovaleryl CoA to methylcrotonyl-CoA, and conversion of methylcrotonyl-CoA to 3-hydroxyisovaleryl-CoA by short-chain enoyl-CoA hydratase (ESCH 1; EC 4.2.1.17)

The branched-chain amino acid leucine has been well studied for its ability to promote positive nitrogen balance as both a protein precursor and a signaling molecule in muscle of rats (Crozier et al. 2004), piglets (Boutry et al. 2013; Yin et al. 2010) and humans (Burd et al. 2012; Churchward-Venne et al. 2012; Li et al. 2011). Leucine has also been shown to decrease muscle protein breakdown in humans (Louard et al. 1990). Leucine serves as a substrate for protein synthesis that occurs in the splanchnic bed and in skeletal muscle. Leucine not incorporated into splanchnic or skeletal muscle protein undergoes complex metabolism yielding several intermediate molecules and is ultimately oxidized (Fig. 1). The first intermediate molecule yielded by leucine metabolism, α-ketoisocaproic acid (KIC), is formed when leucine is reversibly transaminated within the muscle by mitochondrial branched-chain amino transferase (mBCAT). A proportion of KIC is a substrate for branched-chain alpha-keto acid dehydrogenase complex (BCKDC), which irreversibly and oxidatively decarboxylates KIC to form isovaleryl-CoA. Alternatively, a fraction of KIC is decarboxylated and reduced in liver to yield β-hydroxy-β-methylbutyrate (HMB), also known as β-hydroxyisovalerate (CAS 625-08-1). This reaction is catalyzed by ketoisocaproate dioxygenase (4-hydroxyphenylpyruvate dioxygenase; 4-HPPD; EC 1.13.11.27; CAS 9029-72-5) which has been detected in the livers of rats (Sabourin and Bieber 1982) and humans (Xu et al. 2000). In biotin-deficient (Mock et al. 2011) or valproate-treated (Luis et al. 2012) patients, HMB can also be formed from leucine by the “ESCH 1 pathway”, which involves dehydrogenation of isovaleryl-CoA to methylcrotonyl-CoA, and conversion of methylcrotonyl-CoA to 3-hydroxyisovaleryl-CoA by short-chain enoyl-CoA hydratase (ESCH 1; EC 4.2.1.17).

HMB is a metabolite of interest because it has been shown to have some of the anabolic and anti-catabolic effects of leucine on muscle cells, particularly in highly catabolic conditions such as experimentally induced cancer cachexia (Aversa et al. 2011; Smith et al. 2005), bed rest (Deutz et al. 2013), immobilization (Hao et al. 2011), and sepsis (Kovarik et al. 2010; Supinski et al. 2010). In the catabolic state, HMB helps stimulate muscle protein synthesis and inhibit breakdown by reducing proteasome activity and expression of the 20S subunit of the proteasome (Smith et al. 2005), by inhibition of apoptosis (Hao et al. 2011), and by activation of satellite cells (Alway et al. 2013; Kornasio et al. 2009).

Leucine conversion to HMB has been documented in vivo in sheep and pigs (Van Koevering and Nissen 1992) and in humans with biotin deficiency (Mock et al. 2011) or treated with the drug valproate (Luis et al. 2012). However, the metabolic profile of leucine has not been detailed in a rat ADME model. We wanted to explore the absorption, distribution, metabolism, and excretion of leucine using a standard ADME model, the Sprague–Dawley rat. Our study with ^14^C-leucine indicates that conversion to HMB occurs in the rat and is quantifiable in both plasma and urine.

## Methods

### Materials

**Fig. 2:**
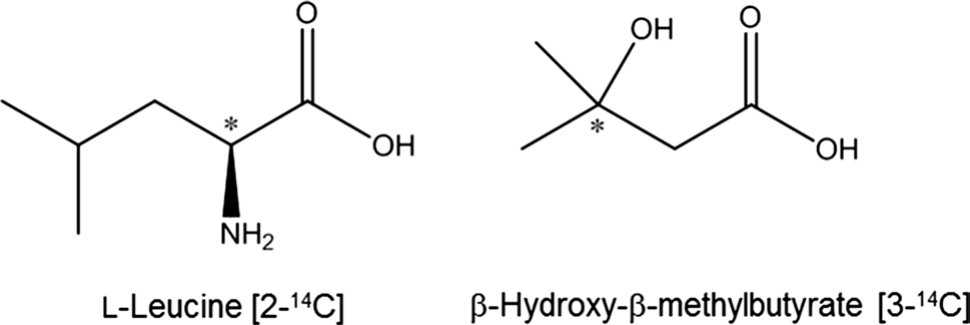
Structure of 2-^14^C-leucine administered to rats and of 3-^14^C-HMB used as an HPLC–MS standard. *Asterisks* denote the location of ^14^C

2-^14^C-l-leucine (55 mCi/mmol) was purchased from American Radiolabeled Chemicals, Inc. (St. Louis, MO) as a 2 % ethanol aqueous solution at 0.1 mCi/mL (Fig. 2). 3-^14^C-β-hydroxy-β-methylbutyrate (HMB) standard was prepared by the Process Organic Chemistry Department (now part of Abbvie, Inc.). In brief, 2-^14^C-acetone (100 mCi at 55 mCi/mmol, American Radiolabeled Chemicals) and ethyl bromoacetate were condensed in a Reformatsky reaction with a Zn–Ag couple. The product, 3-^14^C-HMB ethyl ester, was obtained in 46 % yield and 98 % radiochemical purity. It was saponified to give the 3-^14^C-HMB in 65 % yield and 98 % radiochemical purity as an aqueous solution. A portion of this solution was diluted with water to give the working solution at 1.0 mCi/mL, 98 % radiochemical purity, and 55 mCi/mmol.

### Animals and experimental design

The studies were approved by the Abbott Laboratories (now Abbvie, Inc.) Institutional Animal Care and Use Committee (protocol #1111A0131). A total of twenty-nine 12-month-old male Sprague–Dawley rats (Hilltop Lab Animals, Inc. Scottsdale, PA) were used in these studies and were acclimated to the laboratory for 4 days before use. Rats were given ad libitum access to Harlan Teklad #2018 Rodent Chow and tap water except as noted below for overnight fasting. To minimize animal use, these studies utilized 2–3 rats per group, typical of drug absorption, distribution, metabolism, and excretion studies. Extensive previous experience indicates that this number is adequate to obtain reliable results (Aubert et al. 2012; Gao et al. 2014; Hakk et al. 2009).

Animals were dosed with 3 or 1,000 mg/kg ^14^C-leucine. For the 3 mg/kg dose, a solution of ^14^C-leucine and non-radiolabeled leucine in 5 % dextrose in water containing 1.6 % ethanol was used. This dose was used to for both intravenous and oral administration. The final concentration of this solution was 3 mg/mL and utilized a dose volume of 1.0 mL/kg with a target radioactivity of 50 μCi/rat. For the high-dose studies, a suspension of ^14^C-leucine and non-radiolabeled leucine (1,000 mg/kg total dose) was prepared in a standard oral gavage vehicle, 0.25 % carboxymethylcellulose containing 0.1 % Tween 80. This suspension was used in part due to the limited solubility of leucine at this higher concentration (120 mg/mL) and was different from the vehicle used for the 3 mg/kg dose because only an oral formulation was required. The dose volume for this dose was 8.3 mL/kg and the target radioactivity was 100 μCi/rat. The purity of the formulation under conditions of administration for all dose groups was >97 %.

### Mass balance study

For the mass balance studies, nine male 12-month-old surgically modified rats weighing 593 ± 21 g (mean ± SEM) were used. Rats had cannulas inserted into the bile duct and duodenum as well as a catheter inserted into the femoral artery by Hilltop Lab Animals, Inc., Scottsdale, PA. For this study, the rats were housed individually in metabolism cages (up to 2 days). Rats were fasted overnight and food was returned 4 h post dose. On the day of dosing, the biliary and duodenal cannulas were disconnected and a solution of taurocholic acid (27.8 mg/mL) was infused through the duodenal cannula. Groups of three rats each received a single 3 mg/kg intravenous dose of ^14^C-leucine via the penile vein, a 3 mg/kg oral dose of ^14^C-leucine, or a 1,000 mg/kg oral dose of ^14^C-leucine administered by gavage. Bile, urine, and feces were collected. The cages were washed at the end of study (48 h post dose) with a small amount of 70 % ethanol and the cage wash was collected for determination of total radioactivity.

### Tissue distribution study

For the tissue distribution study, twenty male 12-month-old Sprague–Dawley rats, weighing 633 ± 11 g (mean ± SEM) were fasted overnight before being given a 3 or 1,000 mg/kg (*n* = 10 per group) oral dose of ^14^C-leucine prepared as described above. Subgroups of two rats each were euthanized by 95 % CO_2_–5 % O_2_ at 0.5, 1, 2, 4 and 24 h for collection of blood and selected tissues (plasma, kidney, liver, lung, adipose, muscle, skin, small intestine and stomach). Blood samples were withdrawn via cardiac puncture into EDTA tubes. The plasma samples were obtained by centrifugation of the blood samples.

### General methods

#### Determination of total radioactivity

Total radioactivity in plasma, bile, urine and cage wash was determined directly by liquid scintillation counting (LSC) using a Model 3100TR Quanta Smart Liquid Scintillation Counter (Packard Instrument Company). Feces and tissues were homogenized in 70 % ethanol. Duplicated aliquots of fecal and liver homogenates were combusted in a Tri-Carb^®^ (Packard) Model 307 Sample Oxidizer), followed by LSC. Duplicate aliquots of all other tissue homogenates were solubilized in 1N sodium hydroxide at 40 °C, followed by LSC. Carcass, including gastrointestinal tract and its contents, was weighed and submerged in KOH (10N) overnight on a shaker at low speed. The homogenate was weighed and aliquots were combusted as described above. Total radioactivity was calculated using estimates of total tissue weight (Caster et al. 1956; Davies and Morris 1993).

#### Sample preparation for metabolite identification and profiling

Radioactive materials in plasma were extracted by adding 10 mL of acetonitrile to 1 mL of plasma, followed by vortex mixing and centrifugation. An acetonitrile–methanol mixture (10 mL, 1/1, v/v) was used to extract the remaining non-precipitable radioactive material from the protein pellet. The resulting pellet was then dissolved in 5 mL of 1N sodium hydroxide and incubated for overnight at 60 °C. Radioactivity was assessed at each step of the extraction process by LSC.

#### Analytical methods

Radiolabeled components in urine and plasma samples were profiled following separation by HPLC and detection by an on-line radio flow detector (all samples) and in parallel with a mass spectrometer (Thermo LTQ Orbitrap) for representative samples. The HPLC system consisted of a Thermo Accela 1,250 pump and Thermo Accela autosampler connected to an IN/US β-Ram Model 5 flow scintillation detector equipped with a 500 µL cell. For plasma samples, radioactive profiles were obtained using HPLC fraction collection into Deepwell Luma Plate™-96; the radioactivity was counted by Perkin Elmer Microplate Scintillation Luminescence Counter Topcount NXT.

For plasma samples from rats following the 3 mg/kg dose, the elution of metabolites for negative ion mass spectrometry was achieved at room temperature on a Thermo Syncronis HILIC, 5 µm, 150 × 4.6 mm column equilibrated with 10 % solvent A (ammonium acetate 10 mM) and 90 % solvent B (acetonitrile) at a flow rate of 1.0 mL/min. After 3 min, a linear gradient was run to 40 % solvent A over 14 min and maintained for 2 min. For plasma samples from rats following the 1,000 mg/kg dose and urine samples, the elution of metabolites for further separation of leucine and HMB was achieved at room temperature on the same column equilibrated with 5 % solvent A and 95 % solvent B at a flow rate of 1.0 mL/min. After 5 min, a linear gradient was run to 20 % solvent A over 19 min, followed by a linear gradient to 50 % solvent A over 2 min and maintained for 6 min.

## Results

### Plasma measures

**Fig. 3:**
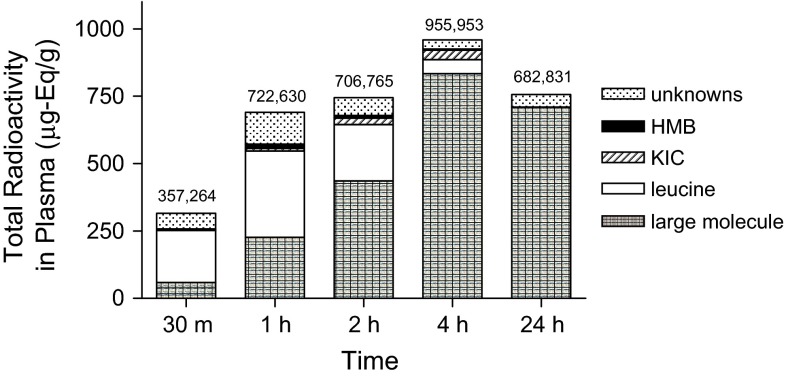
Distribution of ^14^C among leucine metabolites in plasma after oral administration of a bolus of ^14^C-leucine (1,000 mg/kg). *Bar* height is mean of total radioactivity measured in plasma from two rats. Individual data from each rat are presented numerically on top of each *bar*. Pooled plasma from each rat pair was fractionated with acetonitrile (ACN) and the percentage of radioactivity in each fraction assessed. The ACN supernatant was analyzed for leucine, KIC, and HMB

Orally administered ^14^C-leucine appeared in the circulation within 30 min of the gavage and remained detectable for the 24 h time course of the study (Fig. 3). The fate of ^14^C-leucine in plasma following oral dosing of 1,000 mg/kg was investigated by determining the proportion of plasma radioactivity soluble in acetonitrile (ACN). The ACN-insoluble fraction is an estimate of ^14^C-leucine incorporation into plasma protein. Nearly 20 % of plasma ^14^C was found in the ACN-insoluble fraction 30 min post gavage increasing to 60 % by 2 h and to 80 % at 24 h (Fig. 3).

Each ACN-soluble ^14^C-metabolite demonstrated a characteristic time course of appearance in plasma (Fig. 3). While ^14^C-leucine and ^14^C-HMB peaked 1 h after oral dosing, ^14^C-KIC did not appear until 1 h post dosing, and climbed steadily thereafter. The KIC:leucine ratio in plasma ranged from 0 at 30 min to 0.66 at 4 h after the gavage.

**Fig. 4:**
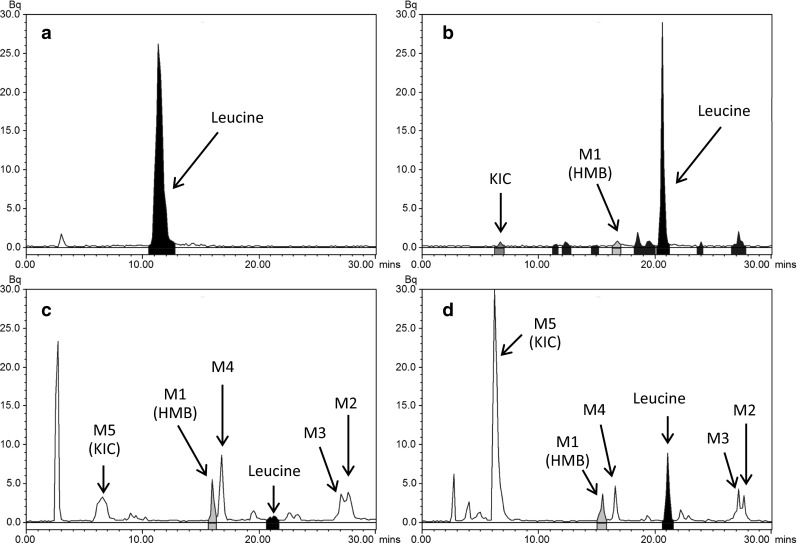
Representative radiochromatograms showing the metabolite profile in plasma (**a**, **b**) and urine (**c**, **d**). Values represent concentration after an oral dose of ^14^C-leucine at 3 mg/kg (**a**, **c**) and 1,000 mg/kg (**b**, **d**). Plasma values were collected 1 h post dose, while urine values were derived from urine collected for 24 h post dose. Slightly different chromatographic conditions were used for the two doses in plasma; validation experiments showing the recovery of spiked leucine and HMB were performed and confirmed identity of the peaks in the figures (data not shown)

The dose of ^14^C-leucine strongly affected metabolite profile in plasma and urine. At 1 h following oral dosing of ^14^C-leucine at 1,000 mg/kg, 73.1 % of the ACN-soluble radioactivity was recovered from plasma as ^14^C-leucine and 3.1 % as ^14^C-HMB (Fig. 4, panel B). In contrast, when ^14^C-leucine was administered orally at 3 mg/kg, 96.3 % of ^14^C was recovered as ^14^C-leucine (Fig. 4, panel A). No peak was detected as ^14^C-HMB in plasma at this dose, even though spike experiments show that HMB could theoretically be recovered under the conditions employed (data not shown). In contrast, urine collected for 24 h following the oral 3 mg/kg dose contained ^14^C-labeled leucine (2.8 %), HMB (7.2 %), KIC (15.0 %) along with other metabolites (Fig. 4, panel C). Following the 1,000 mg/kg dose, 10.7 % of radioactivity in urine appeared as leucine, 4.9 % as HMB, and 53.0 % as KIC (Fig. 4, panel D) along with other metabolites. Spike experiments confirmed the recovery of HMB from urine under the HPLC conditions employed (data not shown).

**Fig. 5:**
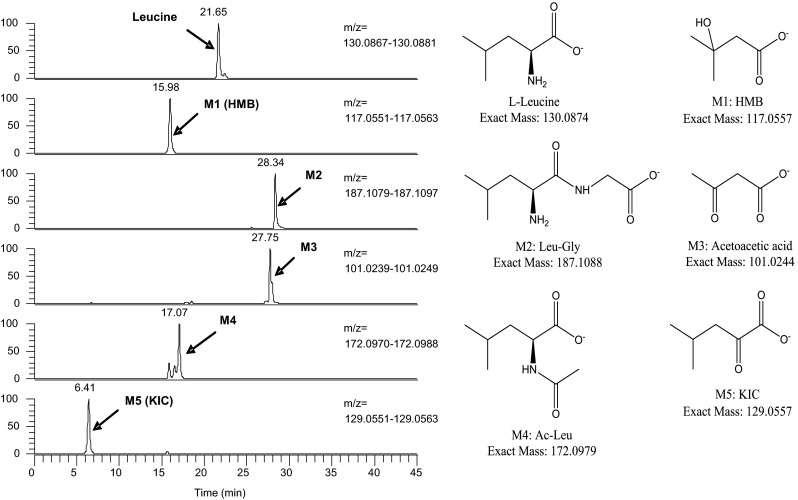
Mass-spectrometric identification of leucine metabolites in urine following a single oral administration of ^14^C-leucine (1,000 mg/kg)

Structural identification of four metabolites (M2, M3, M4, and M5) from the 24-h urine samples following the 1,000 mg/kg oral dose of leucine was completed using LC–MS (Fig. 5). These metabolites were identified as the dipeptides leucyl-glycine (M2; 5.6 % of recovered radioactivity) and *N*-acetyl-leucine (M4; 5.5 %), the ketone body acetoacetic acid (M3; 4.4 %), and the HMB precursor keto-isocaproate (KIC, M5; 53.0 %).

### Mass balance and tissue distribution

When ^14^C-leucine (3 mg/kg) was administered intravenously, 61.4 % was recovered in the carcass 48 h post infusion. No more than 0.9 % of the radioactivity was found in the cage, bile, urine, or feces, respectively. Both the pattern and total percent ^14^C recovery was similar when ^14^C-leucine was given orally at either 3 or 1,000 mg/kg and was similar to that following the 3 mg/kg intravenous dose.

**Fig. 6:**
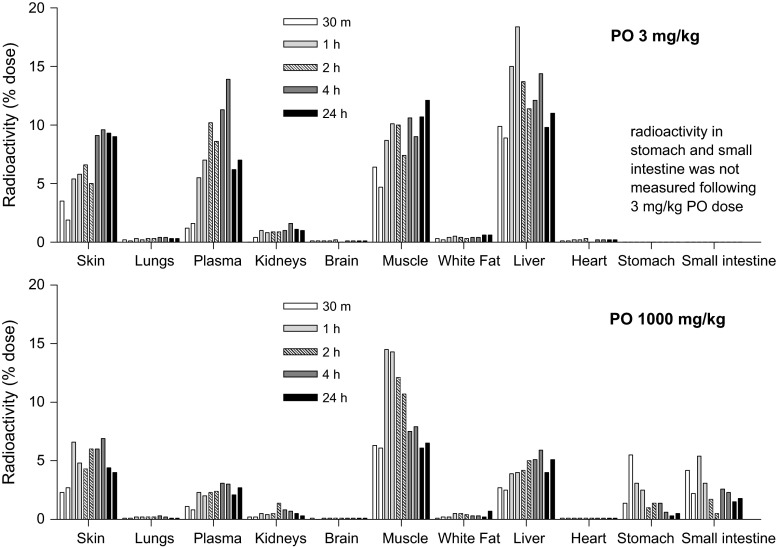
Distribution of ^14^C-leucine among tissues in rats following bolus oral administration of ^14^C-leucine. Each time point represents the percentage of dose administered to an individual rat. There are two *bars* per time point to represent the individual rats from each time point

The time course of tissue distribution of ^14^C varied by dose and by organ (Fig. 6). By total size, skin, skeletal muscle, and liver were the primary targets of ^14^C disposition 24 h post dose with each tissue containing about 10 % of the 3 mg/kg dose or 5 % of the 1,000 mg/kg dose. In these experiments, time points were in duplicate. The figure demonstrates the similarity of the two separate animals for each time point throughout the time course of this study.

## Discussion

The rat ADME model is widely used to predict metabolic products of drugs in humans. Demonstrating that the metabolic products of an amino acid are similar in the rat ADME model to those in human help establish the rat as a valid model for more complex studies. Furthermore, accompanying mass balance studies in rat also indicate the tissue sites associated with metabolism and eventual fate of the radiolabeled molecule. This series of ADME studies uses the rat model to demonstrate for the first time that the essential branched-chain amino acid, leucine, appears in plasma and urine of rats as the metabolite ^14^C-HMB following a dose of ^14^C-leucine. The studies also show for the first time that ^14^C-leucine appears in plasma as ^14^C-keto-isocaproic acid with a slower time course than ^14^C-HMB, its putative product. Further, two novel products of leucine were detected in urine, suggesting additional pathways of leucine metabolism. Interestingly, 2–24 h post dose the majority of circulating ^14^C in the plasma was found in the protein-bound form. The primary organs containing ^14^C at 24 h post dose were muscle, liver, and skin; each contained about 5–10 % of the total ^14^C dose. This study concurs with the previous literature showing that a substantial proportion of administered leucine is oxidized within a few hours (Costelli et al. 1995; White and Brooks, 1981). We found that when a bolus dose of ^14^C-leucine is orally administered to rats, about 35 % is lost from the body after 24 h, suggesting that about one-third of the dose is oxidized to CO_2_.

A surprisingly high proportion of orally administered leucine was retained in plasma even 24 h after administration. This may be partially explained by the observation that over time, ^14^C-leucine (ACN soluble) appeared to be incorporated into plasma protein (ACN precipitable). This is consistent with previous observations that a flooding dose of radiolabeled phenylalanine is incorporated into plasma protein (Papet et al. 2003). Also consistent with the current study, other investigators have found that U-^14^C-leucine is rapidly incorporated into the TCA-insoluble fraction of plasma after intraperitoneal administration in rats (Casati et al. 1979). Therefore, it is possible that a large proportion of the ^14^C-leucine administered may be incorporated into the most abundant plasma protein, serum albumin, allowing the ^14^C-leucine to remain in the circulation since the albumin turnover rate is 62.8 %/day in adult rats (Papet et al. 2003).

After the 1,000 mg/kg dose, ^14^C-HMB appeared in plasma slightly in advance of its putative precursor, ^14^C-KIC. Previous investigators have documented the conversion of ^14^C-leucine to ^14^C-HMB in sheep and pigs (Van Koevering and Nissen 1992), findings we confirm here in a standard preclinical model. ^14^C-KIC appeared in plasma at minute concentrations in the first 2 h after the gavage. By 4 h after the gavage, the ratio of KIC: leucine is about 0.66, similar to that reported by others after an intravenous infusion of 1-^14^C-leucine (Laurent et al. 1984). Interestingly, in our study, the ratio of KIC:leucine derived from a radiolabeled dose is much higher than the ratio (~17 %) of unlabeled molecules reported in the plasma of both fed and starved rats (Hutson and Harper 1981).

The low dose (3 mg/kg body weight) of ^14^C-leucine was chosen to allow comparison of metabolism after oral and IV administration. The high dose (1,000 mg/kg body weight) was chosen to maximize the probability of detecting minor metabolites of leucine. At the 3 mg/kg dose, leucine was the major radiolabeled molecule identified in plasma (96.3 %) and no HMB was detected. However, at this dose, the conversion of leucine to HMB did occur because HMB was identified in the “cumulative” urine collected for 24 h after the dose. In contrast, when ^14^C-leucine was administered at 1,000 mg/kg, over a quarter of the plasma ^14^C was identified as HMB or other metabolites 1 h post gavage. Further examination of the differential response at these two doses demonstrated slightly different patterns of metabolism. In both plasma and urine, the higher dose allowed detection of a greater number of metabolites than the lower dose.

Independent of dose, when ^14^C-leucine was administered either intravenously (IV) or orally, only a small fraction of the ^14^C was recovered from the typical excretion routes of bile, urine (including cage wash) and feces (<5 % total). Because the amount of ^14^C was very low in the feces it is likely that the vast majority of the dose was absorbed. Our data demonstrate that approximately 60–65 % of the dose was recovered in the carcass 48 h post dose leaving the fate of the remaining approximately 35–40 % undetermined. Previous investigators have found that about 8 % of an IV dose of 1-^14^C-leucine is oxidized to CO_2_ by 100 g rats over a 2-h period (Costelli et al. 1995). It is possible that over 48 h, as much as 35 % of orally administered leucine could be lost to complete oxidation to CO_2_.

The major tissues were quantitated for ^14^C at various times up to 24 h post 3 or 1,000 mg/kg oral ^14^C-leucine dose. Regardless of the dose or time following dose, the tissues with the highest ^14^C concentrations were liver, muscle and skin. Previous investigators have also found that ^14^C-leucine and its metabolic products accumulate in liver and muscle after oral administration of ^14^C-leucine (Lemon et al. 1982; Vazquez et al. 1986).

As would likely be expected, there was little ^14^C in the adipose tissue while the lung and kidney demonstrated high per gram concentrations that represented only a small fraction of the total dose when organ size was taken into account. The heart tissue demonstrated higher uptake of the radiolabel than the skeletal muscle on a per gram basis. However, given their relative contribution to the total body weight, skeletal muscle content of ^14^C far exceeded radiolabel in heart. Both muscle and skin were among the lowest concentrations of radioactivity on a per gram tissue basis but accumulated a similar fraction of the total radiolabel as liver. Other investigators have found that even 20 days after the oral dose of radiolabeled leucine, 48 % of the dose was retained, with 3 % in the liver and 8 % in skin (Neale and Waterlow 1974).

In summary, this study has demonstrated that a portion of an oral dose of leucine is converted to HMB in the rat, that HMB may be detectable in urine even when it is not detectable in plasma, and that it appears in plasma in advance of its putative precursor, ^14^C-KIC. A possible explanation for these observations is that both steps of leucine conversion to HMB may occur in some tissues, for instance, kidney. 4-HPPD, the enzyme that converts KIC to HMB, has been found in both liver and kidney as well as in neurons (Neve et al. 2003) and skeletal muscle (Wilkinson et al. 2013). Consequently, local tissue concentrations of HMB may be higher than those measured in plasma. Changes in 4-HPPD activity in physiological states such as aging, cachexia, and diabetes may affect the relative availability of HMB in unsupplemented individuals and should be explored.
